# Canine Geriatric Rehabilitation: Considerations and Strategies for Assessment, Functional Scoring, and Follow Up

**DOI:** 10.3389/fvets.2022.842458

**Published:** 2022-02-25

**Authors:** Christopher Frye, Brittany Jean Carr, Margret Lenfest, Allison Miller

**Affiliations:** ^1^Department of Clinical Sciences, College of Veterinary Medicine, Cornell University, Ithaca, NY, United States; ^2^The Veterinary Sports Medicine and Rehabilitation Center, Anderson, SC, United States; ^3^Department of Biomedical Sciences, College of Veterinary Medicine, Cornell University, Ithaca, NY, United States

**Keywords:** canine (dog), geriatric assessment, function, healthy aging, functional assessment and evaluation, morbidity, rehabilitation, physical therapy

## Abstract

Geriatric animals account for half of the pet population in the United States with their numbers increasing annually. Furthermore, a significant percentage of veterinary patients with movement limitations could be grossly categorized as geriatric and living within the end stage of their predicted lifespans. Because mobility is correlated to quality of life and time to death in aging dogs, a major goal in optimizing canine geriatric health is to improve functional movement. Within the geriatric population, identifying disabilities that affect daily living and quality of life may be used by the rehabilitation practitioner to provide stronger prognoses, treatment goals, and outcome measures. Examples of such means are described within this review. In human medicine, the concept of “optimal aging”, or “healthy aging”, has emerged in which inevitable detrimental age-related changes can be minimized or avoided at various levels of physical, mental, emotional, and social health. Both environment and genetics may influence aging. Identifying and improving environmental variables we can control remain a key component in optimizing aging. Furthermore, diagnosing and treating age related comorbidities common to older populations allows for improved quality of life and is often directly or indirectly affecting mobility. Obesity, sarcopenia, and a sedentary lifestyle are a trifecta of age-related morbidity common to both people and dogs. Healthy lifestyle choices including good nutrition and targeted exercise play key roles in reducing this morbidity and improving aging. Disablement models act as essential tools for creating more effective physiotherapy plans in an effort to counter dysfunction and disability. Within these models, functional testing represents a standard and validated means of scoring human geriatric function as well as monitoring response to therapy. Because of the great need in dogs, this review aims to provide a reasonable and testable standardized framework for canine functional scoring. We believe a complete assessment of canine geriatric patients should comprise of identifying environmental variables contributing to health status; diagnosing comorbidities related to disease and aging; and characterizing disability with standardized methods. Only through this process can we construct a comprehensive, reasonable, and targeted rehabilitation plan with appropriate follow up aimed at healthy aging.

## Introduction

The typical geriatric patient presents to a veterinary rehabilitation service in one of three ways: noted decline in mobility at home; post-surgery physiotherapy; or a decline in mobility noted by another veterinarian. Often times the change in functional movement remains vague until examination and, more often than not, can be attributed to multiple underlying disease processes. Such examples might include an 8-year-old German Shepherd with hip dysplasia, elbow dysplasia, sarcopenia, and degenerative lumbosacral stenosis; or a 10-year-old Labrador Retriever suffering from hypothyroidism, bilateral cranial cruciate disease, copper hepatopathy, and obesity. Although the importance of identifying the major complaint cannot be understated or lost in the complexity of the case, such patients require comprehensive evaluation and therapy for the best outcomes.

As rehabilitation specialists, we attempt to optimize functional movement in our patients. Such an approach is holistic and inevitably is composed of nutritional therapy, pain management, rehabilitative exercises, treatment of co-morbidities, and surgical intervention when indicated. A significant percentage of patients with movement limitations could be grossly categorized as geriatric and living within the end stage of their predicted lifespans (1–3). Within the United States, a more recent census predicted that the geriatric population may amount to nearly 50% of the 78 million owned dogs (1). Given the number of geriatric pets, the relatability of their diseases, and their shared human environment; exploring the role of rehabilitative therapies to optimize geriatric function, quality of life, and longevity warrant discussion and exploration.

The goals of this canine geriatric rehabilitation review are to:

1. Summarize the current pertinent literature and practice
2. Establish a logical, fluid, and comprehensive method for patient assessment, goal setting, and follow up
3. Propose a reasonable and testable framework for standardized functional scoring of the geriatric patient.

## Defining Healthy Aging, Vigor, and Task Dependent Movement

### Healthy Aging

Although there remains a paucity of literature, the natural processes of aging have been studied in dogs and could be considered to be a potential model for human aging (2–6). An individual's life span is influenced by both genetic and environmental factors; however, the length of time one lives often fails to correlate with the quality of life throughout that time. In human medicine, the concept of “optimal aging”, or “healthy aging”, has emerged in which inevitable detrimental age-related changes can be minimized or avoided at various levels of physical, mental, emotional, and social health (7, 8). Healthy aging may be reflected as a delay in the onset of chronic or age-related disease, a reduction in morbidity associated with such disease, an increase in longevity, or any such combination. Although the concept of healthy aging is relatable and understandable, defining the contributing factors and methods to classify a patient (vigor scoring or functional task dependent movement) presents a challenge.

Variables affecting healthy aging in people can be translated to dogs and may include but are not limited to: medical care, social/family support, healthy lifestyle, and environmental conditions (8). These variables may change any time during the lifespan of an animal. Implementing better choices at a younger age may have an even greater impact. As veterinary rehabilitation specialists, we tend to have less influence over establishing better choices in younger healthy dog populations and often encounter our patients for the first time as geriatrics. Regardless of patient age, we often have more success optimizing our patient care if we work with families to identify which variables are impairing the patient and how we can improve them (Table 1).

**Table 1:** Variables affecting healthy aging in dogs.

**Medical care**	**Social/family support**	**Healthy lifestyle**	**Environmental conditions**
Financial resources	Value placed on pet	Appropriate nutrition	Climate/ Season
Geographical access to veterinarian or specialist	Motivation to provide rehabilitative or nursing care	Appropriate and regular exercise	Home layout and potential obstacles or risks
Pet insurance	Physical ability to provide rehabilitative or nursing care	Mental stimulation and engagement	Human and animal interactions (positive or negative)
Temporal access (time of work, childcare, etc.) to see veterinarian	Perspectives on defining a pet's quality of life	Duties or hobbies: sporting, working, therapy, etc.	Other physical enrichment (food puzzles, territorial exploration, access to outdoors, access to shelter, etc.)
Awareness of a problem and where to seek help	Access to resources for or having education in pet care	Preventative care (vaccinations, parasite prevention, dental hygiene, etc.)	Exposure to environmental risks (smoking, pollutants, toxins, infectious or parasitic agents etc.)
Annual or biannual wellness exams			

Adams et al. (2) distinguished *diseases of aging* from the *process of aging*. Various diseases are more likely to occur as an animal ages, especially chronic diseases, and often limit longevity and quality of life. However, all animals experience a *process of aging* in which biochemical and cellular changes lead to progressive senescence of cells and organs and a reduction in functional reserve. Geriatric dogs with mobility issues also commonly present with chronic age-related primary or secondary diseases (for example: diabetes, hypothyroidism, cancer, autoimmune issues, complicated urinary tract infections, etc.). Although it may be too late to prevent such morbidities, many of these diseases are treatable so as to minimize their adverse effects (compression of morbidity) and thereby improve the process of healthy aging (9). Compression of morbidity is not a foreign concept and is regularly employed by veterinarians. As clinicians, we prefer treatments that are curative or near curative, but often must focus on improving circumstances within a pet's remaining lifespan (such as palliative care for appendicular osteosarcoma) or attempting to reduce the rate of a pet's disease progression (such as rehabilitative and nursing care for degenerative myelopathy).

### Vigor

**Figure 1:**
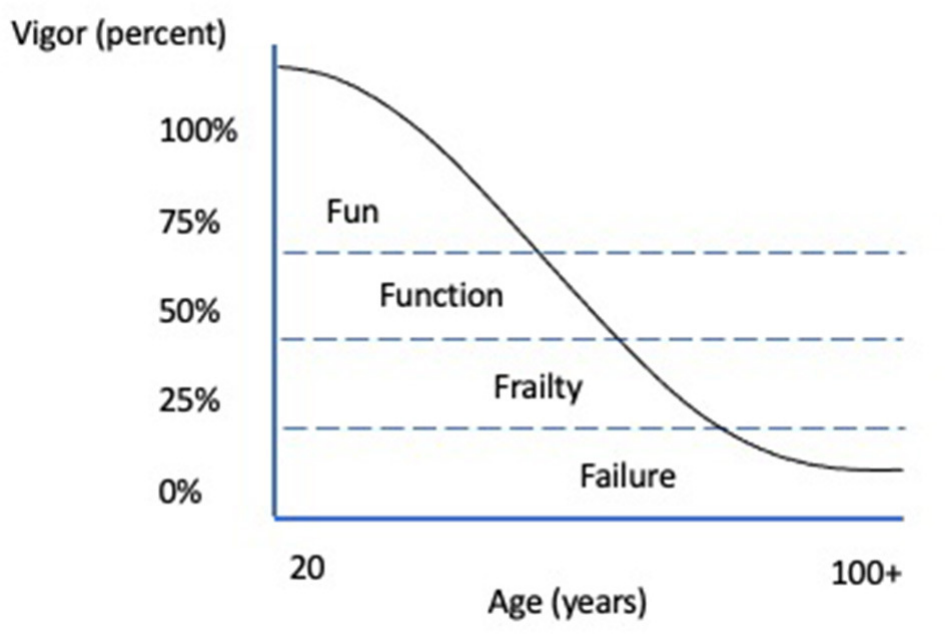
Vigor in Aging People. Adapted from (10).

The concept of “vigor” is an assessment of physical abilities (strength, endurance, balance, spatial awareness, and flexibility), motivation/attitude, comfort, and comorbidities to help further classify daily function and help maximize healthy aging in human geriatric populations (7, 10) (Figure 1). The concept of vigor is logically translatable to aging dogs, but there lacks a validated process to divide aging populations into the progressive categories of fun, functionality, frailty, and failure (Figure 1). Furthermore, unlike people, the breed and size of the dog plays an important role in suggesting the onset of old age with most giant breeds defined as geriatric at 7 years while small breeds take up to 12 years (11, 12). For an individual within any age and canine size cohort, the natural aim would be to maximize the vigor score.

### Task Dependent Movement

Improving functional movement remains at the heart of a canine rehabilitation program. In fact, Hua and colleagues (3) created a canine frailty score based on similar human indices, engaging five components (under-nutrition, exhaustion, low physical activity, poor mobility, and weakness). Poor mobility and low physical activity were both significantly correlated in time to death in this older canine population. Furthermore, a quality-of-life screening program demonstrated that increased activity was the most commonly proposed change to improve canine quality of life (13). The Katz Scale and other derived scales (such as Barthal Index and Lawson and Brody Scale) have been consistently used in human medicine since the mid 20^th^ century to assess one's ability to function independently (14–17). Appropriate task dependent movement relies on a patient's physical abilities, cognition, and motivation. The Katz (15) scale categorizes function into Basic Activity of Daily Living (BADL) whereas Lawton and Brody (17) first identified Instrumental Activity of Daily Living (IADL). BADL includes tasks such as dressing, feeding, and bathing oneself, whereas IADL includes more complicated tasks such as house cleaning, preparing meals, shopping, and managing finances. It has been shown that as independence in function declines from IADL toward BADL, the risk of human hospitalization and death increases (14).

Similar to Katz, we have chosen to define and categorize our geriatric patient function into tasks that are required for: 1. Basic activity for daily independent mobility (BADIM) and 2. Instrumental activities for daily quality of life (IADQOL) (Table 2). We defined the most basic tasks for a dog to maintain independent mobility as rising from a down position; ambulating for short distances including inside and outside the home; posturing to eat or drink; and posturing to eliminate. Examples we have chosen to represent IADQOL are not comprehensive. Regardless, the proposed BADIM and IADQOL classifications aim to assist families and veterinarians into gaining better insight into patient prognosis, establishing more specific rehabilitation goals, and defining and monitoring changes in quality of life (QOL). Research would be required to define ordinal values for each activity and validate them as task dependent movement scales.

**Table 2:** Canine task dependent movement.

**Basic activity for daily independent mobility (BADIM)**	**Instrumental activity for daily quality of life (IADQOL)**
Rising from a down position	Ascending/descending a full flight of stairs
Ambulating in and out of the home	Moving in and out of a vehicle
Posturing to eliminate	Walking short distances outside
Posturing to eat and drink	Exploring the home environment
	Interacting in play (fetch, chase, tug of war, etc.)
	Ability to navigate place of rest (couch, bed, crate, etc.)
	Maintain control of urination and defecation for 6–8 h

## The Syndrome of Age-Related Morbidities: Sarcopenia, Obesity, and Sedentary Lifestyle

Identification and treatment of co-morbidities helps promote better responses to physiotherapy. For example, a Cushingoid dog may suffer from muscle loss, decreased endurance, and degeneration of ligament and tendon structures as part of the disease; all of which impact movement or could contribute to further orthopedic disease like cranial cruciate ligament rupture and Achilles' tendinopathy. A plethora of pathology reducing vigor in aging dogs may be sub-divided by age-related or disease-related morbidities. Three common age-related morbidities described in both human and canine medicine deserve a more in-depth discussion as they influence each other and are commonly present and respond directly to rehabilitation and exercise therapy: sarcopenia, obesity, and sedentary life style. In the experience of these clinicians, it is common to identify all three of these processes together during a geriatric exam. Furthermore, they are all highly inter-related in which an improvement or worsening of one will directly or indirectly impact the other two in similar fashion.

### Sedentary Lifestyle

Subjectively measuring canine activity has proven to be challenging as most dog owners are away from home during the day and inaccurately estimate the duration and intensity of their pet's observed activity (18–20). Both accelerometers and pedometers have been used with success to provide objective data of a pet's daily activity; however, it should be noted that neither method calculates the intensity of that activity (20–23). Although these are useful tools for tracking gross activity, the data may not represent the animal's willingness or ability to exercise as environment (weather, geography, housing conditions, motivating stimuli, etc.), and owner participation/designated time may be the limiting factor. Regardless, both increasing age and increasing body condition have been negatively correlated with activity in dogs (19, 20, 23, 24). It also has been demonstrated that aging dogs, like people, tend to lose lean mass and gain adipose tissue as their metabolic rates decrease (25, 26). Exercise, combined with an appropriate diet, can help combat these changes and potentially delay or reduce the rate of their progression (26, 27). Dogs lacking mobility are predisposed to unwanted sequela including decubital sores, urinary tract infections, skin infections, and pneumonia (28, 29). For these patients, appropriate nursing care, treatment, monitoring, and environmental changes (well-padded clean bedding, floor traction, etc.) are required.

### Sarcopenia

Sarcopenia is the loss of lean mass associated with aging described in many species. It may have multifactorial components including mitochondrial dysfunction, sterile inflammation, hormonal changes, neuronal regulation, and lack of exercise stimulus underlying its pathophysiology (26, 30–32). This disease process alone has been shown to increase morbidity and mortality (3, 26, 31). Lean mass loss during aging has been recorded in dogs (31, 33, 34). Both nutritional and exercise intervention may help reduce the morbidity associated with sarcopenia. Exercise, combining strength training and aerobic activity, may provide the most benefits for both function and muscle mass for people suffering from sarcopenia (26, 30–32). Unfortunately, no such canine research has been conducted; however, Vitger et al. (35) recently demonstrated that exercise preserved lean muscle mass in dogs involved in weight loss programs. Although differences in muscle function, structure, and aerobic capacity exist between people and dogs, the strong parallels regarding sarcopenia in animals allow for reasonable inference that targeted exercise and physiotherapy should also benefit veterinary patients. Diets richer in protein, may also help ameliorate the effects of sarcopenia in otherwise healthy older dogs (36). Furthermore, it has been proposed that geriatrics have a higher protein turnover than their adult counterparts, requiring more protein in their diet to help maintain muscle (37). Therefore, consideration of the quality and quantity of protein in the diet should be optimized whenever possible, taking particular care to maintain daily protein above 3 g/kg lean mass when indicated for dogs on weight loss plans.

### Obesity

It has been consistently shown that obesity decreases longevity and increases morbidity in dogs (19, 23, 26, 27, 38–41). The effects of weight gain on mobility includes exacerbation of osteoarthritis; while weight loss in this canine population has been associated with reduced lameness (42, 43). Furthermore, dogs suffering from obesity that are otherwise healthy, like people, have increases in weight bearing forces and different ranges of motion in their appendicular joints (44). To these authors' knowledge, there has not been research demonstrating worse ambulation or function in obese dogs suffering from neurological disease than those that are lean. However, obesity certainly appears to increase the physical effort and emotional fatigue when rehabilitating non-ambulatory dogs affected by paresis or plegia, both in the clinical setting and in terms of compliance with home care. For example, euthanasia rates are higher for dogs suffering from fibrocartilaginous embolism when they are larger (45). Additionally, geriatric populations may recover more slowly from spinal cord injury (46). Regardless, combatting obesity with appropriate nutritional and exercise intervention can improve movement function, decrease discomfort, and help retain lean muscle mass in aging dogs (27).

## The Disablement Model for Geriatric Assessment, Treatment, Prognosis, and Monitoring

In human medicine multiple models have been developed to both identify and characterize disablement at various levels, most often from the origin, organ/system level, individual person level, and societal/environmental level (47). The benefits of using disablement models include standardizing communication amongst healthcare professionals, providing a contextual framework for directing care based on the unique needs of the patient, and providing an objective tool for research regarding the efficacy and effectiveness of treatments (47, 48). In 1965, the Nagi Disablement Model was developed for humans to describe the impact disease and injury have on an individual at both the level of the person and the level of society (47, 48). The model has four dimensions: active pathology, impairment, functional limitations, and disability. Active pathology is described at the cellular level and defined as damage to the integrity of a body structure. Impairment is described as the abnormality at the tissue, organ, or body system level and includes clinical signs and symptoms. Functional limitations refer directly to the person and are defined by restrictions in performance at the level of the whole person, particularly in relation to the patient's social roles and daily activities. Finally, disability is defined as the inability of the person to fulfill their desired or necessary social or personal roles. If one were to apply this model to a canine patient, Table 3 would be a comparable example for a law enforcement K-9 unit who has sustained a grade II/III iliopsoas tendinopathy (Table 3).

**Table 3:** Nagi model applied to a law enforcement canine patient with a grade II/III iliopsoas tendinopathy.

**Dimension of model**	**Level of disablement**	**Patient example**
Active pathology	Cellular	Grade II/III iliopsoas tendinopathy
Impairment	Body systems	Decreased strength of the iliopsoas, pain upon iliopsoas stretch, decreased flexion/extension of the spine and pelvis
Functional limitations	Whole patient	Inability to extend spine and pelvis when pushing off hind limbs for apprehension work
Disability	Patient's relation to society	Inability to perform apprehension work as K-9 officer

## Functional Testing

Objective methods of monitoring patients are most desirable as they provide a more concrete assessment and prognosis as well as follow up. Many types of objective methods have been previously described elsewhere for dogs and may include muscle girth, kinetic or kinematic gait analysis, weight bearing at a stance, goniometry, and accelerometry/pedometry (49–54). Despite the importance of task dependent movement/function for both owners and veterinarians, a paucity of literature exists in dogs. Measuring and defining function through clinical examination and patient history remains essential to the disablement model process of developing individually targeted physiotherapy. In canine patients, functional limitations are a product of impairment, which can be a decrease in strength, endurance, mobility, balance, proprioception, flexibility, and/or range of motion. Within this context we have reviewed the pertinent canine and human literature; highlighted several methods to examine canine geriatric task dependent movement (within the hospital setting); and proposed a reasonable, testable framework for developing a standardized canine geriatric functional score.

### Strength

In humans, multiple functional tests have been developed as a measure of strength. Decreased strength has been found to correspond to frailty and sarcopenia (55, 56). It has also been correlated to increased risk of fall, morbidity, death, increased hospital stays, and increased hospitalization cost (57–62). The Grip Strength test is a commonly used screening tool that quantifies the maximum force generated by the patient's forearm musculature using a hand-held dynamometer. Unfortunately, this strength test is not applicable to most animals.

Commonly used strength tests to assess lower body strength in humans are the 30 second chair stand test, or the Five Times sit-to-stand (5xSTS). Poor performance on either test has been associated with increased frailty, disability, falls, fractures, and mortality (63–70). Such methods are relatively easy to translate to dogs; however, as dogs are quadrupeds, a more appropriate test may be a sternal recumbent to rise as it engages all weight bearing limbs more equally than a sit to stand (Table 4).

Table 4Canine geriatric functional score tests in order to be administered to patient.

**Table:** 

**Test**	**Test description**	**Scoring**	**Score description**
**A**			
TUG–timed up and go	Rise from down sternal position and move straight (+/– leash) 10 body length units on flat ground with good footing at quickest manageable gait	0	Incapable
		1	> 15 s
		2	>10–15 s
		3	>5–10 s
		4	≤ 5 s
**B**			
Cavaletti	Walk on leash two rails at hock height, body length apart (nose to rump), two rails, two passes (once in each direction) for a total of four rails	0	Incapable
		1	Major contact, navigates slowly with extreme difficulty
		2	Moderate contact, partial gait adjustment
		3	Some contact, adjusts gait accordingly, completes task
		4	Minimal to no contact, navigates well
**C**			
Figure 8's	Figure 8 with diameter of body length for four complete repetitions on leash at a walk	0	Incapable without falling
		1	Consistent knuckling, heavy crossing over, scuffing, delayed pivot
		2	Occasional knuckling, mild to moderate crossing over, scuffing, delayed pivot
		3	Abnormal or delayed pivot (no falls), +/– scuffing
		4	Completes without abnormal crossing over or tripping
**D**			
Down	Sternal to rise until failure within a 60 s period (manual assistance to reposition in sternal allowed)	0	Incapable
		1	<5 reps
		2	>5–10 reps
		3	>10–15 reps
		4	>15 reps

**Table:** 

**Final Summed Score**	**Description**
0–4	Poor
5–8	Fair
9–12	Good
13–16	Excellent

Finally, in human medicine manual muscle testing is used to assess strength on a 1–5 rating scale. These tests require the patient to apply resistance through a body part at different points of range of motion (71) and therefore cannot be applied to dogs. Various forms of canine muscle tests have been described to assess baseline isometric strength in a standing position, such as a timed 3-leg stand (53); however, none have been validated yet. Implementation of such a test in a standard fashion could prove complicated given the uneven distribution of weight between the forelimbs and hindlimbs of a dog.

### Endurance/Mobility

The 6-min walk test was developed to assess mobility and endurance, measuring the distance achieved at a quick walk over 6 min. The 6-min walk test has been used for patients with congestive heart failure, chronic pulmonary disease, and peripheral occlusive arterial disease (72–74). This test was validated to differentiate the difference between healthy dogs and dogs with pulmonary disease (75). Similar outcome measures could be applied to geriatric dogs; however, the duration and physical area required render it less appealing for routine testing.

The “Timed Up and Go” (TUG) Test has been developed to assess human mobility. The patient is observed and timed while they rise from an armchair, walk 3 meters, turn, walk back to the chair, and sit down again. People exceeding 12 s to complete this test are at a greater risk for fall (76–78). While this test has not been validated for dogs, we propose a readily adapted version (Table 4). One would ask the dog to rise from a sternal recumbent position and move 10 body lengths forward at the quickest manageable gait on or off leash. Rising from a sternal position tests weight bearing limbs more evenly than from a sit; furthermore, simplifying the task as a straight line helps reduce variation in following instructions.

Lastly, ratings of perceived exertion (RPE) are used to assess human endurance and conditioning. They are based on an individual's perception of difficulty to perform an exercise or task. These scales have been developed and validated in both adults as well as young children, who often are unable to consistently communicate their feelings regarding exercise (79). Recently a perceived exertion scale (PES) was validated for canine patients and shown to correlate with measured physiologic parameters (80). Dogs were asked to exercise on a treadmill at various intervals for a total time of 55 min. The perceived exertion was recorded every 2 min and rated on a scale of 0 to 4 (Table 5). The study concluded that the PES exhibited consistent and repeatable use when monitoring healthy dogs exercising on a land treadmill at mild to moderate intensity, but that further validation would be required for patients suffering from orthopedic or neurologic disease (80). Such a scale could be easily applied to canine patients receiving regular underwater treadmill therapy when controlling for speed, water height, and inclination by body size. Therefore, we propose walking with the water height at hip level over flat terrain as the best and most accommodating standard test, while applying the same perceived exertion scale as Swanson and colleagues (80). It should be highlighted that results of geriatric dogs partaking in this test may further be confounded by factors beyond conditioning such as pain or neurological status. Unlike Swanson's PES, our proposed test will time a patients' ability to walk before reaching a perceived “moderate effort”. Such a proposal would limit availability to those veterinarians with an underwater treadmill and therefore is less practical or broad reaching when considering development of a standardized universal test. However, further research is warranted to determine if it stands as a valuable tool for some clinicians.

**Table 5:** Canine perceived exertion scale.

**Grade**	**Exertion level**	**Description**
0	No effort noted	No signs of exertion, panting (increased/change in panting), agitation, or abnormal gait
1	Comfortable	May be showing early signs of exertion, very early panting, no to minimal agitation, no change in gait
2	Light effort	Moderate signs of exertion, panting consistently but not labored breathing, mild agitation, no change in gait
3	Moderate effort	Obvious signs of exertion, hard panting, mild labored breathing, moderate agitation, moving slow or reluctantly
4	Significant effort	Obvious signs of exertion, panting very hard, moderate labored breathing, occasional stumbling (<35%)

### Balance/Proprioception

Coordination and proprioception are known to decline in aging human and canine patients. Often a decrease in proprioception and loss of muscle strength can lead to an increased risk for falls and disability (91). In human medicine, several aspects of balance are assessed and categorized into static steady-state balance (maintaining a steady position while sitting or standing), dynamic steady-state balance (walking), proactive balance (anticipating a predicted disturbance such as walking around an obstacle), and reactive balance (compensating for a disturbance) (92). Numerous functional tests have been validated in humans for assessing one or multiple aspects of balance.

The Unipedal Balance Test (UBT) is used to assess static steady-state balance in human patients (93, 94). Another validated and reliable assessment tool for functional balance is the Berg Balance Scale (BBS) with 14 different scaled markers (95, 96). The Dynamic Gait Index (DGI) was developed and validated to assess dynamic balance, rating the ability of the patient to balance while walking and performing eight different tasks (97–100). Finally, the BESTest and Mini-BESTest (shortened version) were developed to assess multiple aspects of balance. The BESTest contains 36 tasks for evaluating 6 different balance control systems, including biomechanical constraints, stability limits with verticality, anticipatory postural adjustments, automatic postural responses, sensory organization, and stability in gait (101). There are currently no validated balance and spatial awareness tests for canine patients. Although three-legged standing tests have been described and are commonly used in rehabilitation practice to assess a canine's strength and steady-state balance (53); this test has a complexity that would likely exclude it from being easily replicated, quantified, or broadly employed. On the other hand, we believe that walking Figure 8's and step over Cavaletti rail obstacles test both the feedforward and feedback systems necessary to judge dynamic balance and spatial awareness with a higher degree of objectivity and less variability amongst patients and practitioners (Table 4).

### Canine Geriatric Functional Score

Similar to human medicine, we believe there remains a dire need to have a validated, practical, and meaningful task-based functional scoring system for our geriatric patients. The Canine Geriatric Functional Score is an assessment tool we are developing to provide an overall measure of function by testing strength, endurance, and balance/spatial awareness through 4 different sequential standardized tasks. The tests should be replicated in a specific order at the beginning of an appointment, as preceding tasks impact latter ones. This assessment is currently undergoing validation trials but can be accomplished in most dogs with relative ease, minimum personnel (two people), and a short time frame (5 min) (Table 4).

## Establishing Reasonable and Achievable Rehabilitation Goals

By engaging the Nagi model, short-term and long-term goals can be derived to address a patient's impairments and functional limits. These goals may need to be modified for the client based on the same variables that effect healthy aging (Table 1). For many geriatric dogs, it is important for clients to understand that patients often will make improvements initially and reach their shorter-term goals; however, other times the rehabilitation process must adjust to maintain such improvements or even reduce the rate of decline. A thorough patient assessment and understanding of optimal aging, can provide the clinician with tools to best convey expectations to owners. Furthermore, appropriately timed follow up remains essential for monitoring patient health status and function and adjusting goals accordingly. As a patient moves into the end stages of life, the rehabilitation specialists should work closely with the family and medical team to identify and establish appropriate quality of life goals, provide palliative and nursing care options, or consider euthanasia.

## Monitoring Progress and Reassessment

Once the Disablement Model has been applied to the geriatric patient, a plan should be made to reach the listed goals within prescribed timeframes. As the canine geriatric patient often has multiple co-morbidities, a team approach (rehabilitation therapist, primary care doctor, other medical specialists, and family) with clear communication is key for successful management.

We recommend initiating more frequent follow ups of the geriatric patient which could include:

- Professional reassessment every 4–6 weeks followed by adjusting the goals and physiotherapy accordingly.
- Baseline and periodic health screening including complete blood count, biochemistry panel, and urinalysis as well as any indicated monitoring or follow-up lab work for metabolic disease or NSAID use.
- Weight and body composition monitoring every 4–6 weeks.
- Regular professional rehabilitative therapy (once to twice weekly when feasible) +/– interventional pain management (such as therapeutic injections, acupuncture, modalities) as indicated.
- A combination of Objective and Subjective Assessment Strategies should be employed.

Consideration in balancing professional clinical rehabilitation with home exercise therapy must be weighed and is often influenced by similar variables to those affecting healthy aging. For example, cost and distance were the two variables most likely to prevent referral of a client seeking a rehabilitation specialist for the pet (102). On the other hand, a study examining outcomes from T3-L3 hemilaminectomies in dogs noted that those receiving rehabilitation had fewer post-operative complications, further supporting the notion that professional physiotherapy allows for closer patient monitoring and timelier intervention (103).

### Objective Assessment Strategies

As reviewed, there are multiple objective assessment strategies the clinician can employ to assess a patient on site. Commonly applied objective assessment strategies used in a clinic setting may include muscle girth, kinetic or kinematic gait analysis, weight bearing at a stance, and goniometry (49–54). It is important to perform these objective assessments at baseline and then every 4–6 weeks (or sooner if there is a change in the patient's status). Collecting the objective measures often may depend on efficiency and availability of materials or trained staff. While objective assessments present a greater challenge to the client at home, monitoring task specific progress is helpful and may include their prescribed home exercise program such as: minutes of leash walking before fatigue, number of sit to stands a patient is able to do in a row or in 30 s, and time the patient is able to hold a 3-legged standing position. Canine digital health monitors may grow in popularity and provide additional information but should be interpreted within context by a professional.

### Subjective Assessment Strategies

The subjective assessment of the patient is often easier for the client to understand, and the information shared is potentially more important to the client. There are a number of validated client specific outcome measure (CSOM) surveys to assess pain and quality of life (Table 6). We engage these surveys for research and occasionally for clinical follow up; however, many clients find them tedious. Despite CSOM value, families also wish to directly discuss how they perceive the patient is doing at home and often have similar markers for their patient assessment. Clients often emphasize the patient's eagerness to move at home or task specific ability such as rising out of bed, roaming the house, navigating out of the home, posturing to eliminate, play, maintaining better traction (reduced slip or fall), and jumping on the bed or into the car. It is important to remember that the family's perspective is vital when determining and discussing quality of life. Ideally a validated task dependent movement scale (such as the proposed BADIM and IADQOL) for companion animals will assist in this process.

**Table 6:** Validated client surveys for canine pain and quality of life assessment.

Canine brief pain inventory (CBPI) (81)
Helsinki chronic pain index (HCPI) (82)
Canine orthopedic index (COI) (83–85)
Liverpool osteoarthritis in dogs (LOAD) (86)
Visual analog scale (VAS) (87)
Glasgow composite measure pain scale short form (CMPS-SF) (88)
Canine health related quality of life survey-21 (CHQLS-21) (89)
Canine osteoarthritis staging tool (COAST) (90)

## Conclusions

The veterinary field lacks standardized scoring systems to assess and better manage an expanding population of geriatric canine patients. Because geriatrics often suffer from mobility issues related to the diseases or processes of aging, they require comprehensive rehabilitative care to optimize their health. Mobility and task specific function are vital to the quality of life in dogs and are prognostic to hospitalization and death in people. Although tools to assess the human patient population have been employed, none are specific to canine geriatrics and none objectively measure function. The complexity of comorbidities associated with aging dogs demands strong communication amongst the care team and family as well as collaborative goal formulation and close follow up. Therefore, we found it imperative to review the available literature; provide a foundation for canine geriatric assessment, goal setting, and follow up; and propose a testable framework for geriatric specific functional scoring. Furthermore, we hope this review springboards ideas for canine geriatric specific rehabilitative research.

## Author Contributions

All authors listed have made a substantial, direct, and intellectual contribution to the work and approved it for publication.

## Conflict of Interest

The authors declare that the research was conducted in the absence of any commercial or financial relationships that could be construed as a potential conflict of interest.

## Publisher's Note

All claims expressed in this article are solely those of the authors and do not necessarily represent those of their affiliated organizations, or those of the publisher, the editors and the reviewers. Any product that may be evaluated in this article, or claim that may be made by its manufacturer, is not guaranteed or endorsed by the publisher.
